# Cytotoxic T lymphocyte‐associated antigen‐4 (CTLA-4) gene polymorphisms in a cohort of Egyptian patients with immune thrombocytopenia (ITP)

**DOI:** 10.1007/s44313-024-00011-z

**Published:** 2024-03-01

**Authors:** Doaa Mohamed El Demerdash, Maha Mohamed Saber, Alia Ayad, Kareeman Gomaa, Mohamed Abdelkader Morad

**Affiliations:** 1 Internal Medicine Department, Faculty of Medicine, Teaching Kasr AL-Ainy Hospital, Cairo University, Al Kasr Al Aini, Old Cairo, 4240310 Cairo Governorate Egypt; 2 Clinical and Chemical Pathology Department, Faculty of Medicine, Kasr AL-Ainy Hospital, Cairo University, Cairo, Egypt

**Keywords:** ITP, SNPs, Immune thrombocytopenia, Immune checkpoints, CTLA-4

## Abstract

**Background:**

Immune thrombocytopenia (ITP) is characterized by immune response dysregulations. Cytotoxic T lymphocyte‐associated antigen‐4 (CTLA‐4) plays a central role in immune checkpoint pathways and preventing autoimmune diseases by regulating immune tolerance. We aimed to explore the potential association between CTLA-4 gene polymorphisms and ITP as well as study their impact on the response to therapy.

**Methods:**

We investigated two CTLA-4 single‐nucleotide polymorphisms (SNPs; rs: 231775 and rs: 3087243) using real-time PCR as well as the plasma levels of CTLA-4 by ELISA in 88 patients with ITP and 44 healthy participants (HC).

**Results:**

CTLA-4 (rs: 3087243) A > G polymorphism analysis showed most HC had the homozygous AA genotype, which was statistically significant compared to patients with ITP. Plasma levels of CTLA4 were statistically lower in patients with acute ITP. There was no correlation between CTLA-4 (rs: 231775 and rs: 3087243) A/G SNPs were not correlated to the response to all lines of therapy assessed (corticosteroids, thrombopoietin receptor agonists, splenectomy, and rituximab).

**Conclusion:**

CTLA-4 CT 60 A/G may affect the susceptibility of ITP, but both CTLA-4 + 49 A/G and CT60 A/G did not impact the response of patients with ITP to different lines of therapy.

## Introduction

Immune thrombocytopenia (ITP), one of the most common acquired bleeding disorders, is characterized by reduced platelet count and increased bleeding risk [1].

Several immune mechanisms, including increased platelet destruction in the reticuloendothelial system and disturbed platelet production in the bone marrow, contribute to ITP pathogenesis. However, the exact pathogenesis of ITP is not clear yet [2]*.*

Although antibody-mediated platelet destruction is a well-known primary immunologic defect, ITP pathogenesis also involves a hyper-activated T-cell response, which is important for cell-mediated platelet destruction [3]. Therefore, investigating T-cell abnormalities in patients with ITP may resolve the mechanism of pathogenesis and behavior of ITP as well as the diverse response of therapies.

The immune checkpoint pathways are critical modulators of the immune system, allowing immune response initiation and preventing autoimmunity onset. Immune checkpoints, including co-stimulation and co-inhibition signal pathways, are among the central mechanisms that regulate T-cell-mediated immune responses [4].

A "co-stimulation" signal, which is provided by CD28 on T-cells engaged with B7 family members on antigen-presenting cells (APCs), is necessary for efficient T-cell activation [5].

Cytotoxic T lymphocyte‐associated antigen‐4 (CTLA‐4) is a CD28 structural homolog, a co-inhibitory molecule expressed by activated cytotoxic T-cells, and a specific surface marker of T-reg, which plays a central role in immune checkpoint pathways. CTLA‐4 has a higher affinity for B7 than CD28 and is responsible for T-cell inactivation. Therefore, CTLA‐4 has a role in preventing autoimmune diseases by regulating immune tolerance [6].

CTLA‐4 production is strongly influenced by genetic factors. Single‐nucleotide polymorphisms (SNPs) of the CTLA-4-encoding genes are involved in the pathogenesis of many autoimmune diseases such as systemic lupus erythematosus (SLE) and rheumatoid arthritis (RA) [7]. To explore the potential role of CTLA‐4 in the pathogenesis of primary ITP, we aimed to explore the potential association between single gene polymorphisms of CTLA-4 and primary ITP as well as study their impact on the response to therapy.

## Patients and methods

### Patients

We enrolled 88 Egyptian patients with primary ITP as well as 44 age- and sex-matched unrelated Egyptian individuals living in the same geographical region as healthy participants (HC) from the clinical hematology unit, Internal Medicine Department of the Kasr Al-Ainy teaching hospital. They were diagnosed according to the criteria of the ITP International Working Group (IWG) and followed up prospectively between September 2021 and March 2023 [8]. Patients with disorders possibly associated with secondary thrombocytopenia were excluded from the study. We conducted a single-institution case–control study. Informed consent was obtained from all patients before study initiation and patient recruitment, the study was approved by the local ethical committee (MD-225–2021). The study complied with good clinical practice protocols and with the ethical rules stated in the Declaration of Helsinki (as revised in Tokyo 2004).

### Methods

All participants with ITP as well as HCs were subjected to full history taking (particularly bleeding phenotypes, drug intake, family history, recent immunizations as well as Obstetrics history) thorough clinical examination and laboratory investigations, including complete blood count (CBC) & film, reticulocyte count, and erythrocyte sedimentation rate (ESR). Patients with thyroid diseases or secondary ITP due to viral infections (hepatitis B virus, hepatitis C virus, human immunodeficiency virus), drugs, *Helicobacter pylori* infection, and autoimmune diseases such as SLE were excluded.

ITP was defined as isolated thrombocytopenia (platelet count < 100 × 10^3^/µL) with no evidence of another underlying disorder.

All patients when indicated for therapy received corticosteroids (oral prednisolone 1 mg/kg/day) as the first line of therapy for maximum 21 days and gradually withdrawn. The patients who failed the first line of therapy received second line therapies such as thrombopoietin receptor agonists (TPO-RAs; eltrombopag), monoclonal anti (CD20) rituximab, or splenectomy. Some patients may fail one line and shift to another line (receive more than one line of therapy). The choice of the second line of therapy was according to patient preference, local availability, and physician choice.

Response to therapy was assessed after three months of therapies as follows:

- Complete response (CR): Platelet count ≥ 100 × 10^3^/µL and absence of bleeding.
- Response (R): Platelet count ≥ 30 × 10^3^/µL and at least a twofold increase in the baseline count and absence of bleeding.
- No response (NR): Platelet count < 30 × 10^3^/µL or less than twofold increase of baseline platelet count or bleeding [9].

### Genotyping

Genomic DNA was extracted from whole blood collected on EDTA using a DNA extraction kit (Qiagen GmbH, Hilden, Germany) according to the manufacturer's protocol. Real-Time PCR with sequence-specific primers was used to assess the CTLA-4 genotype (+ 49 A/G rs: 231,775 and CT60 A/G rs: 3,087,243). Variants were determined using real-time PCR and high-resolution melt analysis on the CFX95 real-time system C 1000 thermal cycle (Biorad). The context sequence for CTLA-4 (+ 49 A/G rs: 231,775) was: forward primer: (5-GCTCTACTTCCTGAAGACCT-3 and reverse primer: (5-AGTCTCACTCACCTTTGCAG-3), The context sequence for CTLA-4 (CT60 A/G rs: 3,087,243) was: forward primer: (5′-ATAATGCTTCATGAGTCAGCTT-3′) and a reverse primer: (5′-GAGGTGAAGAACCTGTGTTAAA-3′). All reactions were performed in 20 μL reaction mix containing 10 μL master mix, 0.5 μL SNP-readymade assay, 1–5 μL purified DNA solution according to DNA concentration, completed to 20 μL with nuclease-free water.

Steps of performing PCR and the thermal cycling conditions.

**Table:** 

PCR (40 Cycles)		AmpliTaq Gold Enzyme Activation
Anneal/Extend	Denature	HOLD
1 min at 60 °C	15 s at 95 °C	10 min at 95 °C
Specify the reaction volume (20 μL/well) in a 48-well plate		
Load the reaction plate into the thermal cycler, and then start the run		

Relation between fluorescence signals and sequences in a sample.

**Table:** 

Indicates	A substantial increase in
Homozygosity for Allele 2	FAM-dye fluorescence only
Homozygosity for Allele 1	VIC-dye fluorescence only
Allele 1- Allele 2 heterozygosity	Both VIC- and FAM-dye fluorescence

After PCR amplification, an endpoint plate read was performed using a DNA-Technology Real-Time PCR System. The DT master Software used the fluorescence measurements made during the plate read to plot fluorescence values based on the signals from each well. The plotted fluorescence signals indicated which alleles were in each sample. The plate-read document was analyzed. Automatic allele calls were made. Allele calls were converted to genotypes.

### Statistical analysis

Data was collected, tabulated, and statistically analyzed using an IBM-compatible personal computer with Statistical Package for the Social Sciences (SPSS) version 26. The quantitative data are presented in the form of mean, standard deviation (SD), median, and range, and qualitative data were presented in the form of numbers (N) and percentages (%). Chi-square test (χ^2^) or Fisher’s Exact test were used to study the association between two qualitative variables, Student’s t-test (t) was used to compare the quantitative variables between two groups of normally distributed data, Mann–Whitney U test was used to compare the quantitative variables between two groups of non-normally distributed data.

Results were considered statistically significant at *P* < 0.05.

## Results

### Clinical and laboratory features

In this case–control study, the median age of the patients with ITP and HCs was 32.5 (15–50) and 30.5 (15–46) years, respectively. Moreover, most of the patients with ITP were females (73.9%). The median disease duration was 5.4 (0–11) years. Regarding the clinical presentation, no patients had a history of NSAID use or herbal supplement intake and all patients presented with mucocutaneous bleeding in the form of epistaxis, purpura, or vaginal bleeding. Only one patient (1.1%) presented with severe bleeding (internal bleeding) and another patient (1.1%) had a history of previous repeated abortions, but no history of thrombotic events. None of the patients had lymphadenopathy or splenomegaly. The mean platelet count at diagnosis was 17.2 × 10^3^/µL ± 12.5 (2–60 × 10^3^/µL). Since all studied patients had primary ITP, the virological screening was negative for all patients. Autoimmune screening, including anti-nuclear antibody (ANA) and anti-dsDNA, of the patients was negative. Screening for antiphospholipids was also negative and only one patient had subclinical hypothyroid. The clinical and demographic characteristics as well as response of patients with ITP to therapies are summarized in Table 1.

**Table 1:** The clinical characteristics of patients with ITP (N, 88)

**Parameter**		**Subcategory**	**Frequency**	**Percentage**
**Sex**		M	23	26.1
		F	65	73.9
**Bleeding score**		0	7	7.9
		1	67	76.1
		2	15	17
**Type of bleeding**		Muco-cutaneous	79	89.7
		gastrointestinal	7	8.5
		Genitourinary	38	43
		Internal bleeding	1	1.1
**ITP Phase**		Newly diagnosed	4	4.5
		Persistent	11	12.5
		Chronic	37	42
**Number of lines of therapies**		1	4	4.5
		2	59	67
		3	18	18
		4	7	8
**Steroid dependency**		Dependent	52	63.4
		Non-dependent	30	36.6
**Second line therapies**		Yes	84	48.7
		No	4	51.2
**Response to therapies**	**Corticosteroids (N, 88)**	CR	8	9.1
		R	64	72.7
		NR	16	18.2
	**TPO-RAs (N, 67)**	CR	18	26.9
		R	33	49.3
		NR	16	23.9
	**Rituximab (N, 38)**	CR	10	26.3
		R	16	42.1
		NR	12	31.6
	**Splenectomy (N, 11)**	CR	4	36.4
		R	4	36.4
		NR	3	27.3

*ITP* Immune thrombocytopenia, *N* Number, *TPO-RAs* Thrombopoietin receptor agonists, *M* Male, *F* Female, *CR* Complete response, *R* Response, *NR* No response

### Response to therapies

Briefly, 88 (100%) patients received corticosteroids as the first line of therapy. Among them, 8 (9.1%) patients had a complete response (CR). The patients received second line therapies when they failed to respond to or were dependent on the first line of therapy to maintain platelet response. Some patients may fail one line and be shifted to another line (received more than one line of therapy). A total of 67 and 38 patients received eltrombopag and anti (CD20) rituximab, respectively, of whom 18 (26.9%) and 10 (26.3%), respectively, had a CR. Additionally, 11 patients underwent splenectomy and 4 (36.4%) of them had CR. Unfortunately, the duration of maintaining CR in each line was not documented.

### Genotyping of CTLA-4

The two CTLA-4 gene SNPs (+ 49A/G rs231775 and CT60 A/G rs3087243) were assessed. The CT 60 A/G rs:3087243 (*P* = 0.001) was significantly different between patients with ITP and HCs, while SNP + 49A/G rs:231,775 was not significantly different between patients with ITP and HCs (Table 2).

**Table 2:** Genotypes of CTLA-4 rs231775 and rs3087243 in Patients with ITP and HC

SNPs	Genotype	Cases (%)N, 88	HC (%)N, 44	*P-value*
**rs231775**	**GG**	44 (50)	15 (34.1)	0.206
	**GA**	35 (39.8)	24 (54.5)	
	**AA**	9 (10.2)	5 (11.4)	
**rs3087243**	**GG**	23 (26.1)	9 (20.5)	0.001*
	**GA**	31 (35.2)	0 (0)	
	**AA**	34 (38.6)	35 (79.5)	

^*^Significance difference (*P* < 0.05); CTLA-4: cytotoxic T lymphocyte‐associated antigen‐4; ITP: immune thrombocytopenia; HC: healthy participants; SNPs: single‐nucleotide polymorphisms; N: number

We further investigated the correlation between CTLA-4 genotypes (+ 49A/G rs; 231,775 and CT60 A/G rs: 3,087,243) and the response to different lines of therapy; however, it was not statistically significant in our studied patients with ITP (Table 3).

**Table 3:** Correlation between Genotypes of CTLA-4 rs231775 and rs3087243 and Response to Therapies in Patients with ITP

Line of therapy	Response	rs231775 cases (%)			*P-value*	rs3087243cases (%)			P-value*
		**GG**	**GA**	**AA**		**GG**	**GA**	**AA**	
**Corticosteroids** **(N, 88)**	**CR**	5 (11.5)	3 (8.6)	0 (0)	0.948	2 (8.7)	4 (12.9)	2 (5.9)	0.861
	**R**	32 (72.5)	25 (71.4)	7 (77.8)		16 (69.6)	22 (71)	26 (76.5)	
	**NR**	7 (15.9)	7 (20)	2 (22.2)		5 (21.7)	5 (16.1)	6 (17.6)	
**TPO-RAs** **(N, 67)**	**CR**	8 (25)	6 (22)	4 (50)	0.437	3 (18.8)	9 (36)	6 (23.1)	0.676
	**R**	15 (46.9)	16 (59.3)	2 (25)		8 (50)	12 (48)	13 (50)	
	**NR**	9 (28.7)	5 (18.5)	2 (25)		5 (31.1)	4 (16)	7 (26.9)	
**Rituximab** **(N, 38)**	**CR**	6 (31.6)	3 (20)	1 (25)	0.926	2 (16.7)	6 (54.5)	2 (13.3)	0.129
	**R**	8 (42.1)	6 (40)	2 (50)		5 (41.7)	2 (18.2)	9 (60)	
	**NR**	5 (26.3)	6 (40)	1 (25)		5 (41.7)	3 (27.3)	4 (26.7)	
**Splenectomy** **(N, 11)**	**CR**	1 (25)	2 (33.3)	1 (100)	1	1 (25)	1 (50)	2 (40)	1
	**R**	2 (50)	2 (33.3)	0 (0)		2 (50)	1 (50)	1 (20)	
	**NR**	1 (25)	2 (33.3)	0 (0)		1 (25)	0 (0)	2 (40)	

*CTLA-4* Cytotoxic T lymphocyte‐associated antigen‐4, *ITP* Immune thrombocytopenia, *TPO-RAs* Thrombopoietin receptor agonists, *N* Number, *CR* Complete response, *R* Response, *NR* No response; *Significance difference (*P* < 0.05)

## Discussion

Several immune mechanisms contribute to ITP pathogenesis and interact to affect the response to therapy. The involvement of all T-cell subtypes in ITP pathogenesis has been explored for many years [10–12]. CTLA-4 is a competitive antagonist for B7 on the surface of APCs and responsible for T-cell inactivation and immune tolerance owing to its involvement in the immune checkpoint pathways [13]. The SNPs of CTLA-4-encoding genes are involved in the pathogenesis of many autoimmune diseases [7]. However, the association between the immune checkpoint pathway and ITP pathogenesis is still unclear.

We investigated the impact of CTLA-4 SNPs [+ 49A/G (rs231775) and CT60 A/G (rs3087243)] on the susceptibility and response to therapy in primary ITP for finding new clues in the pathogenesis of primary ITP.

A total of 88 patients with ITP (male:female = 21%:79%) and 44 HCs were enrolled. Although there were conflicting reports regarding sex predominance, our results come in agreement with those of many studies reporting that ITP is common in young and middle-aged females [14–16], while few reports reporting that it is common in older men [17, 18].

To the best of our knowledge, few studies have addressed the influence of SNPs of CTLA-4 in ITP (Table 4). However, this is the first study to find a significant difference in CT60 A/G (rs3087243) genotype between patients with ITP and HCs as most HCs had AA genotypes and lack heterozygous GA genotypes, which may be correlated to the susceptibility of ITP. Moreover, we did not find a difference in + 49 A/G (rs231775) genotype between patients with ITP and HC. Interestingly, *Kasamatsu *et al. did not find a difference in + 49 A/G and CT60 A/G genotype between patients with ITP and HCs, but reported a correlation between CTLA4 CT60 GG genotype (low expression type) and severe clinical presentation at diagnosis while studying four CTLA-4 SNPs in 119 patients with chronic ITP [19]. Moreover, Chen et al., studied nine SNPs of the promoter region of CTLA-4 in 32 patients with ITP and showed no difference in + 49 A/G and CT60 A/G genotype between patients with ITP and HC, while a different SNP (rs11571315) was a susceptible SNP for primary ITP risk in the Taiwanese population [20]. *Yao *et al. studied CT60 A/G (rs3087243) genotype in 102 Chinese Han pediatric patients with ITP and showed that the genotypes were not significantly different between the pediatric patients with ITP and healthy participants. However, they also found decreased CTLA4 expression in patients with ITP, supporting that CTLA4 gene expression instead of gene mutation is one of the factors for ITP [21].

**Table 4:** Immune checkpoint-related gene polymorphisms in ITP

Reference	N	Sex	Mean age (Y)	Studied polymorphisms	Conclusions
Wang et al., [22]	307	F 62% M 38%	44.92 ± 14.02	**TIM3** rs10515746 **CD28** rs1980422 **TNFSF4** rs2205960 **CTLA4** rs231779 **PD1** rs36084323 **ICOS** rs6726035 **DNAM1** rs763361 **LAG3** rs870849	**CD28 rs1980422** was associated with an increased risk of ITP after false discovery rate correction (*P* = 0.006) The T allele of **PD1 rs36084323** was a risk factor for ITP severity and the T allele of **DNAM1 rs763361** for corticosteroid-resistance The combination of **TIM3 rs10515746**, **CD28 rs1980422**, and **ICOS rs6726035** best predicted a high risk of ITP
Chen et al., [20]	32	F 84.5% M 15.5%	60 (median)	12 SNPs analysis mainly focused on the promoter region of **CTLA-4** and **CD28**	**CTLA-4 rs11571315** was a susceptible SNP for primary ITP in the Taiwan population **CTLA-4 rs5742909** was related to secondary ITP caused by autoimmune disease **CTLA4** was associated with ITP but not **CD28**
Kasamatsu et al., [19]	119	F 73% M 27%	64 (median)	**DCD1 SNPs:** (606 G/A, + 7209 C/T, + 63379C/T) **CTLA4 SNPs:** (1722 A/G, 1577 A/G, + 49 A/G, CT60 A/G)	**PDCD1 + 7209 C/T** SNP is associated with susceptibility to cITP no significant differences in **CTLA4 SNPs** between cITP patients and healthy individuals **CTLA4 1577 A/G and CT60 A/G** polymorphisms may affect the severity of cITP
Aktürk et al., [23]	62	NA	NA	**CTLA-4 A49G**	no association between the **A49G** polymorphism of the **CTLA-4 gene** and ITP
Yao et al., [21]	102	F 70.7% M29.3%	5.43 ± 3.21 (pediatric)	**CTLA-4** rs11571315 and rs3087243	**CTLA4 gene** is suggested to correlate with ITP through its abnormal expression level instead of gene site mutation
Li et al., [24]	186			**CTLA-4** 318 and CT60	these two SNPs in **CTLA-4** are not associated with susceptibility to ITP in a Chinese population
Radwan et al., [25]	100	F 58% M 42%	7.3 ± 4.4 (pediatric)	**CTLA-4** exon1 49 A > G	It did not detect a relation between the selected **CTLA-4** SNP and susceptibility to ITP in Egyptian children
Our presented research	88	F 74% M 26%	32.5 (median)	**CTLA-4** rs: 231,775 rs: 3,087,243	**CTLA-4 rs: 3,087,243** may affect the susceptibility of ITP but there was no impact of both studied SNPs (**rs: 231,775 and rs: 3,087,243) of CTLA-4** on the response to different lines of therapy in adult ITP patients

*ITP* Immune thrombocytopenia, *CTLA-4* cytotoxic T lymphocyte‐associated antigen‐4, *N* number; %: percentage, *Y* years, *M* male, *F* female, *NA* None applicable; *Significance difference (*P* < 0.05)

The conflicting results may be due to different studied ethnic populations, disease stages, or the potential role of epigenetic modifiers that may need more exploration in future studies.

Although we did not find a correlation regarding CTLA-4 + 49 A/G (rs231775) between patients with ITP and HCs, a large metanalysis by *Wang *et al., who searched the same SNP in different autoimmune diseases like RA and type 1 DM including a considerable number of patients (N, 4732), found that CTLA-4 + 49 G/A (rs231775) is associated with the susceptibility of autoimmune disease in Asian and Caucasian populations [26].

Pavkovic et al*.* studied CTLA-4 + 49 A/G in different hematological diseases (AIHA, ITP, and CLL) and found no difference between patients with ITP (N*,* 60) and healthy participants, but the G allele of CTLA-4 predisposes to AIHA development, particularly among patients with CLL [27].

Wang et al*.* studied different immune checkpoint-related gene polymorphisms including CTLA-4 (rs231779) in 307 patients with ITP and stated that immune checkpoint-related SNPs, particularly CD28 rs1980422, may be genetic factors associated with ITP development and treatment. However, neither allelic nor genotypic frequencies of CTLA4 rs231779 were significantly associated with the susceptibility to or severity of ITP [22].

In this study, we did not find any correlation between the two CTLA4 SNPs and the response to all therapies including corticosteroids, TPO-RAs, splenectomy, and rituximab in our studied patients. To the best of our knowledge, very few studies have assessed the correlation between CTLA-4 SNPs and the response to therapy in ITP, particularly the response to corticosteroids. This is the first study to assess this correlation with different lines of therapy but a bigger sample size of patients in each group of therapy may be needed. Kasamatsu et al*.*, found no correlation between CTLA4 polymorphisms and the response to prednisolone therapy and splenectomy in 52 patients with ITP [19]. Moreover, Wang et al*.,* found that the allelic or genotypic frequencies of CTLA4 (rs231779) were associated with corticosteroid sensitivity of ITP [22]. Zhu et al*.* monitored plasma levels of CTLA-4 in 37 patients with ITP who received 40 mg/day dexamethasone for four consecutive days and found that CTLA-4 levels were significantly elevated in not only patients with acute ITP, but also responders with acute ITP, suggesting that CTLA-4 might be associated with the pathogenesis of acute ITP and reflect treatment efficacy [28]. Moreover, Guo et al*.,* observed dynamic changes in CTLA-4 and CD28 expression after high-dose dexamethasone therapy in 28 patients with ITP, suggesting that a disturbed CD28/CTLA-4 balance may contribute to ITP immunopathogenesis [29].

## Conclusion

CTLA-4 CT 60 A/G may affect the susceptibility of ITP, but both CTLA-4 + 49 A/G and CT60 A/G had no impact on the response to different lines of therapy in patients with ITP. Thus, further studies should include more patients with ITP in each group of therapy as well as focus on more immune checkpoint-related gene polymorphisms.

## Data Availability

All data generated or analyzed during this study are included in this published article.

## Abbreviations

ANA: Anti-nuclear antibody
APCs: Antigen-presenting cells
CBC: Complete blood count
cITP: Chronic immune thrombocytopenia
CR: Complete response
CTLA-4: Cytotoxic T lymphocyte‐associated antigen‐4
DM: Diabetes mellitus
DNA: Deoxyribonucleic acid
EDTA: Ethylenediaminetetraacetic acid
ESR: Erythrocyte sedimentation rate
HC: Healthy participants
ITP: Immune thrombocytopenia
IWG: International Working Group
N: Number
NR: No response
PCR: Polymerase chain reaction
R: Response
RA: Rheumatoid arthritis
SLE: Systemic lupus erythematosus
SNPs: Single‐nucleotide polymorphisms
SPSS: Statistical Package for the Social Sciences
TPO-RAs: Thrombopoietin receptor agonists
